# The Relationship Between Dynamic Changes in the Insulin Resistance–Related Indices and Metabolic Syndrome in Middle‐Aged and Elderly Population

**DOI:** 10.1155/ije/9331905

**Published:** 2026-03-03

**Authors:** Xinfeng Li, Xiaohui Li, Chifa Ma, Chenfei Li, Mingxia Yuan

**Affiliations:** ^1^ Department of Endocrinology, Beijing Friendship Hospital, Capital Medical University, Beijing, China, ccmu.edu.cn

**Keywords:** area under the curve, dynamic changes, insulin resistance, metabolic syndrome

## Abstract

**Background:**

Insulin resistance is the central pathogenesis of metabolic syndrome. Insulin resistance–related indices have been shown to identify the metabolic syndrome. The present study aims to explore the predictive value of four insulin resistance–related indices for the metabolic syndrome and the association between dynamic changes in these indices and the metabolic syndrome.

**Methods:**

3,526 participants aged ≥ 45 years were enrolled from the China Health and Retirement Dynamic Study. After a 4‐year follow‐up, 761 participants developed metabolic syndrome. The receiver operating characteristic curve was used to evaluate the predictive value. The restricted cubic spline was used to explore the presence of a nonlinear relationship between indices and metabolic syndrome. Logistic regression was used to analyze the dynamic changes in insulin resistance indices in the metabolic syndrome.

**Results:**

TyG–BMI and Mets‐IR have larger AUC. TyG–BMI, TG/HDL‐c, and Mets‐IR exhibit a nonlinear association with the metabolic syndrome. Participants with low–high and high–high variability patterns have an increased risk of metabolic syndrome. For TG/HDL‐c, the high–low pattern is also associated with a higher risk of developing metabolic syndrome.

**Conclusions:**

TyG–BMI and Mets‐IR could be better indices for predicting metabolic syndrome in middle‐aged and elderly populations. For individuals with indices below the cutoff points, it is advisable to avoid an increase in IR‐related indices to prevent metabolic syndrome. A dynamic variety of insulin resistance–related indices could predict a higher risk of the incidence of metabolic syndrome.

## 1. Introduction

Metabolic syndrome is defined as a disorder of energy metabolism and the collection of multiple metabolic risks, including obesity, insulin resistance (IR), hypertension, and dyslipidemia [1]. Metabolic syndrome increases the risk of Type 2 diabetes, cardiovascular disease, and all‐cause mortality. It is also associated with thrombotic and inflammatory states, nonalcoholic fatty liver disease, and reproductive disorders [2]. Studies have shown that nearly one in five adults in the Asia‐Pacific region suffers from metabolic syndrome and the number of sufferers continues to rise [3]. It is essential to explore simple and practical clinical indicators, which may help to predict the risk of metabolic syndrome in the early stage and make appropriate prevention strategies.

The core pathophysiological mechanisms of metabolic syndrome are IR and dyslipidemia [4]. Relevant meta‐analysis indicates that individuals with higher IR exhibit a higher incidence of metabolic‐related diseases [5]. The degree of individual IR could assess and predict the occurrence of metabolic syndrome [6]. The gold standard for clinically assessing IR is the hyperinsulinemic euglycemic clamp. However, this evaluation method is complex, expensive, and invasive, making it unsuitable for widespread clinical application [7]. Currently, several clinically useful surrogate measures, such as the ratio of triglyceride/high‐density lipoprotein cholesterol (TG/HDL‐c), the metabolic score of insulin resistance (Mets‐IR), and the TG–glucose index in conjunction with body mass index (TyG–BMI), have been shown to be effective for assessing IR degree [8]. The IR‐related indices have been shown to be widely and accurately used to assess IR, comparable to the traditional index HOMA‐IR [9]. These measures, based on routine physical examination results, could be more economical and convenient for assessing IR.

TyG, TyG–BMI, Mets‐IR, and TG/HDL‐c have been shown to identify metabolic syndrome in the cross‐sectional study [10, 11]. TyG and its derived indices have been shown to be more accurate in identifying metabolic syndrome [12]. A meta‐analysis that includes 13 observational studies shows that patients with metabolic syndrome have higher levels of TyG. TyG is more capable of identifying metabolic syndrome, with the area under the summary receiver operating characteristic (ROC) curve of 0.87 in the cross section [13]. In addition, the ability of TyG to predict metabolic syndrome in the long term has been demonstrated [14]. However, there are fewer cohort studies of TyG–BMI, Mets‐IR, and TG/HDL‐c in the prediction of metabolic syndrome. Remnant cholesterol (RC) is a lipid‐related indicator that includes very low‐density lipoprotein (VLDL), intermediate‐density lipoprotein (IDL), and chylomicron, which has been found to be associated with adverse cardiovascular events and IR [15, 16], but the relationship between RC and metabolic syndrome is not yet clear.

Previous research types have mainly focused on the ability of IR‐related indices to identify and predict metabolic syndrome, which mostly use transient indices to assess and predict metabolic syndrome [17]. Fewer studies have explored the relationship between dynamic changes in IR‐related indices and the metabolic syndrome. However, metabolic syndrome is a long‐term disorder of energy metabolism. Studying the dynamics of the relevant indices could more accurately predict metabolic syndrome and observe the effect of index trends on the metabolic syndrome. The present study is a nationwide prospective cohort study targeting the middle‐aged and elderly population in China based on the data from the China Health and Retirement Longitudinal Study (CHARLS). This study examines the association between dynamic changes in four alternative markers of IR and the risk of metabolic syndrome. Additionally, we evaluate the predictive ability of the novel index RC for the metabolic syndrome.

## 2. Methods

### 2.1. Data Source

The data are downloaded from the CHARLS, which is a publicly available, high‐quality, nationally representative database that provides a comprehensive range of information on middle‐aged and older adults and their families [18]. The CHARLS receives approval from the Ethical Review Committee at Peking University, and all participants sign informed consent forms. The following information is collected from CHARLS: basic demographics, family, health status and function, cognition and depression, health care and insurance, work and retirement, pensions, income expenditure and assets, housing, and blood data including HbA1c, fasting plasma glucose (FPG), and lipid profile (TG, HDL‐c, total cholesterol).

### 2.2. Study Population

CHARLS has completed five years of follow‐up since 2011, involving a total of 17,708 individuals and their spouses, with follow‐up intervals of every two to three years. Since the present study requires blood test data, we have selected a population that was followed up on from 2011 to 2015. The final inclusion criteria are aged 45 and above, and individuals are free from metabolic syndrome at baseline and complete demographic data, including marriage, age, education level, and complete blood test results, including fasting blood glucose, lipid profile, and C‐reactive protein. A total of 3526 individuals are eligible for the current study.

### 2.3. Measurement and Metabolic Syndrome Diagnostic Criteria

According to the physical examination questionnaire on the official website of CHARLS (http://CHARLS.pku.edu.cn/en), the physical examination of the participants was measured by professional staff using a uniform type of equipment. Blood pressure was measured by staff using Omron sphygmomanometers, and participants were instructed to have their palms facing upward, with their left arm released and their left arm at the same level as their heart. Height was measured using vertical height scales, and weight was measured using weight thumps by staff. Waist circumference was measured by a staff member using soft measuring tapes at the end of the participant’s breath hold.

According to the blood testing manual on the official CHARLS website, participants were asked to fast on the day and their blood specimens were collected by professional Chinese Center for Disease Control and Prevention (China CDC) staff. These specimens were then immediately stored frozen at −20°C and transported to the China CDC in Beijing within 2 weeks, where they were placed in a deep freezer and stored at −80°C until assay at the Chinese Medical University laboratory. TG, FPG, and HDL‐cwere tested at the Capital Medical University Youanmen Clinical Laboratory using the enzyme colorimetric method.

Four IR‐related indicators are used to identify metabolic syndrome [19–22]:

(1) TyG−BMI=Lnfasting TGmg/dL×FPGmg/dL/2×BMIkg/m2,

(2) TGHDL−c=fasting TGmg/dLfasting HDL−c cholesterolmg/dL,

(3) Mets−IR=Ln2×FPGmg/dL+fasting TGmg/dL×BMIkg/m2LnHDL−cmg/dL,

(4) RC=TCmg/dL−LDL−cmg/dL−HDL−cmg/dL.

Definition of metabolic syndrome according to the Chinese criteria of the NCEP ATP III (2005) [23, 24]: (1) The waist circumference defining central obesity is ≥ 80 cm for women and ≥ 90 cm for men. (2) Elevated TG levels refer to TG levels ≥ 150 mg/dL or specific treatment for this lipid abnormality. (3) Low HDL‐c levels refer to HDL‐c cholesterol < 40 mg/dL in males,  < 50 mg/dL in females, or specific treatment for this lipid abnormality. (4) Elevated BP is indicated by systolic BP > 130 or diastolic BP > 85 mm Hg, or treatment of previously diagnosed hypertension. (5) Elevated FPG levels are defined as FPG levels ≥ 100 mg/dL or the use of antidiabetic medications or self‐reported medical history of diabetes. Metabolic syndrome could be diagnosed when at least 3 of the 5 items are present.

### 2.4. Assessment of Covariates and Group of Index Dynamic Changes

The present study includes covariates based on the following three criteria: 1. variables with significant p‐values in single‐factor analysis; 2. variables that cause the initial regression coefficients to change by more than 10% in stepwise regression models; 3. variables associated with metabolic syndrome in clinical practice. According to the data from CHARLS regarding demographics, health function‐related questionnaires, and anthropometric measurements, the following factors are adjusted: 1. Demographics: age, gender, education level (including illiteracy, elementary school, high school, and above college), and marital status (married or single). 2. Health function‐related questionnaires: smoking status (nonsmokers or smokers), alcohol consumption status (nondrinkers or drinkers), and frequency of meals (less than three meals a day, three meals, or more than three meals a day). 3. Anthropometric measurements: creatinine and C‐reactive protein. To analyze the changes in TG/HDL‐c, TyG–BMI, Mets‐IR, and RC throughout follow‐up, participants are classified into high and low levels based on the thresholds derived from the analysis of restricted cubic spline curves. The thresholds for TG/HDL‐c, Mets‐IR, TyG–BMI, and RC are defined as 1.55, 44.19, 172.85, and 19.33, respectively. The change in each IR surrogate during the follow‐up period from 2011 to 2015 is categorized into four patterns: (1) Low–low: consistently low levels during follow‐up; (2) low‐to‐high: low levels at baseline but changed to high levels at follow‐up; (3) high‐to‐low: high levels at baseline but changed to low levels at follow‐up; (4) high–high: consistently high levels throughout follow‐up.

### 2.5. Statistical Analysis

Continuous variables are tested for normality using the Shapiro–Wilk test. Categorical variables are expressed as percentages. Normally distributed data are expressed as mean and standard deviation (SD) for continuous variables, while non‐normally distributed data are expressed as median (interquartile range, IQR). Students’ *t*‐test, chi‐square test, and the Wilcoxon rank‐sum test are used to examine the baseline characteristics and prevalence of metabolic syndrome. The predictive power of different indices for the onset of metabolic syndrome is investigated using the ROC curve, and the area under the curve (AUC) is calculated. The AUC of different indices is compared using the DeLong test. The restricted cubic spline is used to explore the presence of a nonlinear relationship between different indices and metabolic syndrome. The likelihood ratio test is used to explore the cutoff point of the relationship between indices and metabolic syndrome. Four IR‐related indices are considered as continuous variables, which are positioned at the 5th, 35th, 65th, and 95th percentiles. Furthermore, the effect of dynamic changes in different indices on the metabolic syndrome is investigated. Logistic regression is used to calculate the odds ratio (OR) for the low‐to‐high, high‐to‐low, and high‐to‐high, with the low‐to‐low as the reference.

The statistical analysis is performed using R software (Version 4.2.0) and the statistical software EmpowerStats (http://www.empowerstats.net/cn/index.php). The package “plotRCS” is used for the analysis of restricted cubic splines. A level of p < 0.05 is considered statistically significant.

## 3. Results

### 3.1. Characteristics of Participants

Table 1 shows the characteristics of the participants in 2011 and 2015. A total of 3526 middle‐aged and older participants without metabolic syndrome are included in this study, with a median participant age of 58 years in 2011. There are a total of 1933 (54.91%) male and 1587 (45.09%) female participants, with a mean BMI of 22.16 kg/m^2^ and a median WC of 80 cm. The median of RC, Mets‐IR, TyG–BMI, and TG/HDL‐c in the total population was 15.08, 47.76, 182.53, and 1.55, respectively. After 4 years, all the above indicators have increased.

**TABLE 1:** Characteristics of study participants in 2011 and 2015.

Variable	Total in 2011 (*n* = 3526)	Total in 2015 (*n* = 3526)
Age, median (*Q*_1_, *Q*_3_)	58.00 (52.00, 65.00)	62.00 (56.00, 69.00)
Gender, *n* (%)		
Male	1933 (54.91)	1933 (54.91)
Female	1587 (45.09)	1587 (45.09)
WC (cm), median (*Q*_1_, *Q*_3_)	80.00 (75.00, 86.00)	81.80 (75.20, 88.00)
BMI (kg/m^2^), mean ± SD	22.16 ± 3.39	22.49 ± 3.83
SBP (mmHg), mean ± SD	123.82 ± 28.66	126.74 ± 46.06
DBP (mmHg), mean ± SD	72.50 ± 11.85	73.51 ± 11.85
Creatinine (mg/dL), mean ± SD	0.78 ± 0.18	0.82 ± 0.29
CRP (mg/dL), median (*Q*_1_, *Q*_3_)	0.80 (0.47, 1.72)	1.20 (0.70, 2.30)
Mets‐IR, median (*Q*_1_, *Q*_3_)	47.76 (42.74, 53.56)	49.27 (43.37, 55.85)
TyG–BMI, median (*Q*_1_, *Q*_3_)	182.53 (165.60, 200.95)	187.36 (167.58, 210.18)
TG/HDL‐c, median (*Q*_1_, *Q*_3_)	1.55 (1.08, 2.23)	1.84 (1.31, 2.76)
RC, median (*Q*_1_, *Q*_3_)	15.08 (8.89, 22.81)	23.17 (18.15, 30.12)

*Note:* (*Q*_1_, *Q*_3_), interquartile range; TG/HDL‐c, TG/HDL‐c; TyG–BMI, TG–glucose index with body mass index.

Abbreviations: BMI, body mass index; CRP, C‐reactive protein; DBP, diastolic blood pressure; Mets‐IR, metabolic score for insulin resistance; RC, remnant cholesterol; SBP, systolic blood pressure; SD, standard deviation; WC, waist circumference.

Table 2 shows the characteristics of the study population at baseline, depending on whether or not one has the metabolic syndrome. After 4 years of follow‐up, a total of 761 (21.58%) of the 3526 individuals developed metabolic syndrome. Metabolic syndrome patients have higher BMI, creatinine, and C‐reactive protein levels, and the TyG–BMI, Mets‐IR, RC, and TG/HDL‐c indices are also higher at baseline. Nonmetabolic syndrome individuals with lower smoking and drinking rates might be related to self‐regulation based on individual health changes. Metabolic syndrome is more prevalent in women.

**TABLE 2:** The characteristics of the study participants were grouped by metabolic syndrome at baseline.

Variable	Total (*n* = 3526)	Occurrence of metabolic syndrome		*P*
		NO (*n* = 2765)	Yes (*n* = 761)	
Age, median (*Q*_1__1_, *Q*_3_)	58.00 (52.00, 65.00)	58.00 (52.00, 65.00)	57.00 (51.00, 64.00)	0.099
Gender, *n* (%)				< 0.001
Male	1933 (54.91)	1616 (58.55)	317 (41.71)	
Female	1587 (45.09)	1144 (41.45)	443 (58.29)	
Marita, *n* (%)				0.085
Nonmarried	371 (10.52)	278 (10.05)	93 (12.22)	
Married	3155 (89.48)	2487 (89.95)	668 (87.78)	
Education status, *n* (%)				0.011
Illiterate	964 (27.34)	722 (26.11)	242 (31.80)	
Primary school and below	2247 (63.73)	1797 (64.99)	450 (59.13)	
Middle school	228 (6.47)	181 (6.55)	47 (6.18)	
Frequency of meals, *n* (%)				0.922
< Three meals	498 (14.12)	391 (14.14)	107 (14.06)	
Three meals	2952 (83.72)	2313 (83.65)	639 (83.97)	
> Three meals	76 (2.16)	61 (2.21)	15 (1.97)	
Smoking, *n* (%)				< 0.001
Nonsmoker	1594 (45.21)	1324 (47.88)	270 (35.48)	
Smoker	1932 (54.79)	1441 (52.12)	491 (64.52)	
Drinking, *n* (%)				< 0.001
Nondrinker	1055 (29.92)	865 (31.28)	190 (24.97)	
Drinker	2471 (70.08)	1900 (68.72)	571 (75.03)	
WC (cm), median (*Q*_1_, *Q*_3_)	80.00 (75.00, 86.00)	79.00 (74.20, 84.50)	84.60 (78.00, 90.00)	< 0.001
BMI (kg/m2), mean ± SD	22.16 ± 3.39	21.74 ± 3.22	23.69 ± 3.52	< 0.001
SBP (mmHg), mean ± SD	123.82 ± 28.66	122.62 ± 25.45	128.22 ± 37.86	< 0.001
DBP (mmHg), mean ± SD	72.50 ± 11.85	71.83 ± 11.63	74.96 ± 12.34	< 0.001
Creatinine (mg/dL), mean ± SD	0.78 ± 0.18	0.78 ± 0.19	0.76 ± 0.18	< 0.001
CRP (mg/dL), median (*Q*_1_, *Q*_3_)	0.80 (0.47, 1.72)	0.78 (0.46, 1.66)	0.93 (0.56, 1.85)	< 0.001
Mets‐IR, median (*Q*_1_, *Q*_3_)	47.76 (42.74, 53.56)	46.52 (41.84, 51.82)	52.37 (47.12, 58.88)	< 0.001
TyG–BMI, median (*Q*_1_, *Q*_3_)	182.53 (165.60, 200.95)	178.39 (162.26, 195.85)	197.50 (181.54, 216.89)	< 0.001
TG/HDL‐c, median (*Q*_1_, *Q*_3_)	1.55 (1.08, 2.23)	1.46 (1.01, 2.10)	1.92 (1.34, 2.60)	< 0.001
RC, median (*Q*_1_, *Q*_3_)	15.08 (8.89, 22.81)	14.30 (8.51, 22.42)	17.40 (10.82, 25.13)	< 0.001

*Note:* (*Q*_1_, *Q*_3_), interquartile range. Mets‐IR, metabolic score for insulin resistance; TG/HDL‐c, triglyceride/high‐density lipoprotein cholesterol; TyG–BMI, triglyceride–glucose index with body mass index.

Abbreviations: BMI, body mass index; CRP, C‐reactive protein; DBP, diastolic blood pressure; RC, remnant cholesterol; SD, standard deviation; SBP, systolic blood pressure; WC, waist circumference.

### 3.2. Relationship Between Baseline IR‐Related Index and Metabolic Syndrome

Table 3 shows the relationship between the four assessment indices and the onset of metabolic syndrome by means of multifactorial regression models. The risk of metabolic syndrome increases in a linear trend with increasing RC, Mets‐IR, TyG–BMI, and TG/HDL‐c. In the RC group, using the lowest quartile as a reference, there is no statistically significant difference in the second quartile (OR = 1.23; 95%CI:0.96, 1.58, *p* = 0.116), and the prevalence of metabolic syndrome increases in the third and fourth quartiles, respectively (OR = 1.72; 95%CI: 1.35, 2.20) (OR = 2.05; 95%CI: 1.61, 2.62). In terms of the other three indicators, using the lowest quartile as a reference, the second, third, and fourth quartiles have progressively higher risks of metabolic syndrome.

**TABLE 3:** Relationship between 4 IR‐related indices and risk of metabolic syndrome.

Variables	Model 1	*P*	Model 2	*P*	Model 3	*P*
Mets‐IR	1.07 (1.06, 1.08)	< 0.001	1.08 (1.07, 1.09)	< 0.001	1.08 (1.07, 1.09)	< 0.001
Quartiles of Mets‐IR						
1	1.00 (Reference)		1.00		1.00	
2	2.36 (1.73, 3.20)	< 0.001	2.57 (1.88, 3.51)	< 0.001	2.60 (1.90, 3.56)	< 0.001
3	3.81 (2.84, 5.10)	< 0.001	4.33 (3.20, 5.86)	< 0.001	4.37 (3.23, 5.92)	< 0.001
4	7.67 (5.77, 10.18)	< 0.001	9.19 (6.82, 12.38)	< 0.001	9.34 (6.92, 12.60)	< 0.001
p for trend		< 0.001		< 0.001		< 0.001
TyG–BMI	1.02 (1.02, 1.02)	< 0.001	1.02 (1.02, 1.03)	< 0.001	1.02 (1.02, 1.03)	< 0.001
Quartiles of TyG–BMI						
1	1.00 (Reference)		1.00		1.00	
2	2.02 (1.48, 2.75)	< 0.001	2.15 (1.57, 2.93)	< 0.001	2.18 (1.59, 2.99)	< 0.001
3	3.82 (2.86, 5.10)	< 0.001	4.15 (3.09, 5.59)	< 0.001	4.19 (3.11, 5.64)	< 0.001
4	7.65 (5.78, 10.12)	< 0.001	8.58 (6.39, 11.50)	< 0.001	8.70 (6.47, 11.68)	< 0.001
p for trend		< 0.001		< 0.001		< 0.001
TG/HDL‐c	1.35 (1.26, 1.45)	< 0.001	1.43 (1.33, 1.54)	< 0.001	1.44 (1.33, 1.54)	< 0.001
Quartiles of TG/HDL‐c						
1	1.00 (Reference)		1.00		1.00	
2	1.58 (1.21, 2.07)	< 0.001	1.51 (1.15, 1.98)	0.003	1.51 (1.15, 1.98)	0.003
3	2.44 (1.89, 3.15)	< 0.001	2.40 (1.86, 3.11)	< 0.001	2.41 (1.86, 3.12)	< 0.001
4	3.66 (2.86, 4.69)	< 0.001	4.02 (3.13, 5.18)	< 0.001	4.06 (3.15, 5.23)	< 0.001
p for trend		< 0.001		< 0.001		< 0.001
RC	1.02 (1.02, 1.03)	< 0.001	1.03 (1.02, 1.03)	< 0.001	1.03 (1.02, 1.03)	< 0.001
Quartiles of RC						
1	1.00 (Reference)		1.00		1.00	
2	1.23 (0.96, 1.57)	0.104	1.24 (0.97, 1.59)	0.090	1.23 (0.96, 1.58)	0.116
3	1.68 (1.32, 2.13)	< 0.001	1.73 (1.35, 2.21)	< 0.001	1.72 (1.35, 2.20)	< 0.001
4	1.95 (1.54, 2.47)	< 0.001	2.07 (1.62, 2.63)	< 0.001	2.05 (1.61, 2.62)	< 0.001
p for trend		< 0.001		< 0.001		< 0.001

*Note:* Model 1 is a crude model. Model 2 is adjusted for age, gender, education level, married status, and frequency of meals. Model 3 is additionally adjusted for smoking, drinking, creatinine, and C‐reactive protein.

### 3.3. Predictive Power of the IR‐Related Index for the Metabolic Syndrome

**FIGURE 1:**
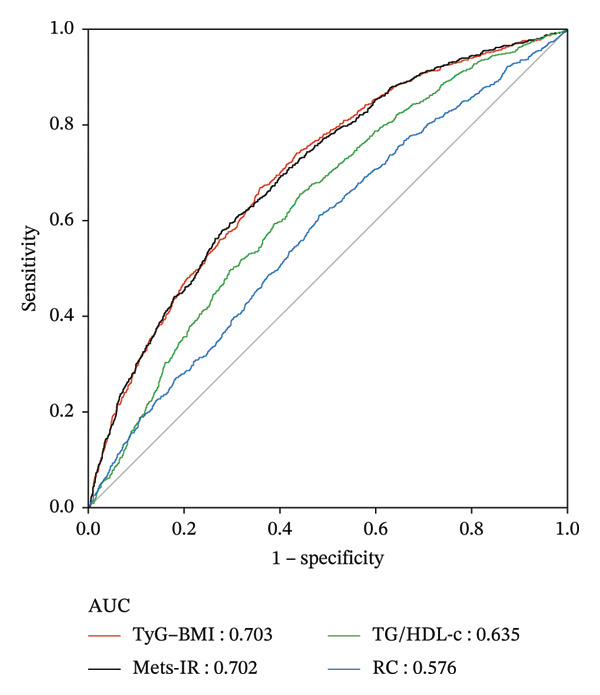
Receiver operating characteristic curves of four indices (TyG–BMI, Mets‐IR, TG/HDL‐c, RC).

Figure 1 represents the predictive value of the four indices for metabolic syndrome. The highest AUC value is TyG–BMI (AUC = 0.703). The predictive abilities of the other indices are Mets‐IR (AUC = 0.702), TG/HDL‐c (AUC = 0.635), and RC (AUC = 0.576). Table 4 (the DeLong test) indicates the difference of four indices in predicting the incidence rate of metabolic syndrome. TyG–BMI and Mets‐IR have a better ability compared to the other two indices (*p* < 0.001). RC has a poorer ability (*p* < 0.001). TyG–BMI and Mets‐IR have no statistically significant difference in their abilities to predict the incidence of metabolic syndrome (*p* = 0.7773).

**TABLE 4:** The difference of four index abilities in predicting the incidence rate of metabolic syndrome.

Variables	AUC (SE) (95%CI)	^-∗^Mets‐IR (95%CI)*p*	^-∗^TyG–BMI (95%CI)*p*	^-∗^TG/HDL‐c (95%CI)*p*
Mets‐IR	0.7015 (0.0105) (0.6808, 0.7222)			
TyG–BMI	0.7028 (0.0105) (0.6821, 0.7234)	0.0012 (−0.0074, 0.0099) 0.7773		
TG/HDL‐c	0.6345 (0.0110) (0.6129, 0.6561)	0.0670 (0.0435, 0.0905) < 0.0001	0.0683 (0.0444, 0.0921) < 0.0001	
RC	0.5759 (0.0117) (0.5529, 0.5988)	0.1257 (0.0986, 0.1527) < 0.0001	0.1269 (0.1009, 0.1529) < 0.0001	0.0587 (0.0406, 0.0767) < 0.0001

*Note:* *p* is calculated by using the method DeLong test comparing the AUC of four indices (TyG–BMI, Mets‐IR, TG/HDL‐c, RC).

^-∗^The difference between the two indices.

### 3.4. Restricted Cubic Spline

In Figure 2, to demonstrate the relationship between metabolic syndrome and IR‐related indices more clearly, we present the dose–response relationship using restricted cubic spline bars. Four IR‐related indices are significantly associated with metabolic syndrome. TyG–BMI, TG/HDL‐c, and Mets‐IR exhibit a nonlinear association with the metabolic syndrome (*p* < 0.001, *p* for overall nonlinearity < 0.001), with a lower prevalence below their respective thresholds (172.85, 1.55, and 44.19). RC shows a linear relationship with the onset of metabolic syndrome, with a threshold of 19.33 for its onset.

FIGURE 2Restricted cubic spline modeling of four indices adjusted for risk of metabolic syndrome. (a) TG/HDL‐c indicator. (b) TyG–BMI indicator. (c) Mets‐IR indicator. (d) RC indicator. Adjustment variables: sex, age, education level, married status, frequency of meals, smoking, drinking, creatinine and C‐reactive protein.

**Figure:**
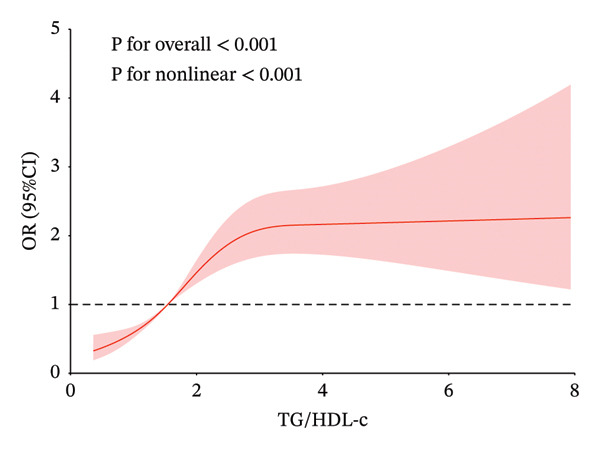
(a)

**Figure:**
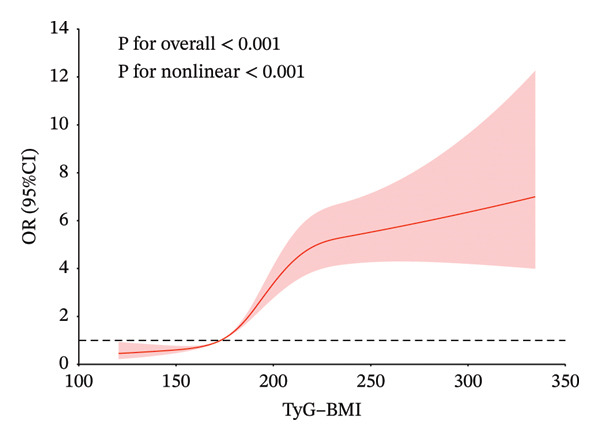
(b)

**Figure:**
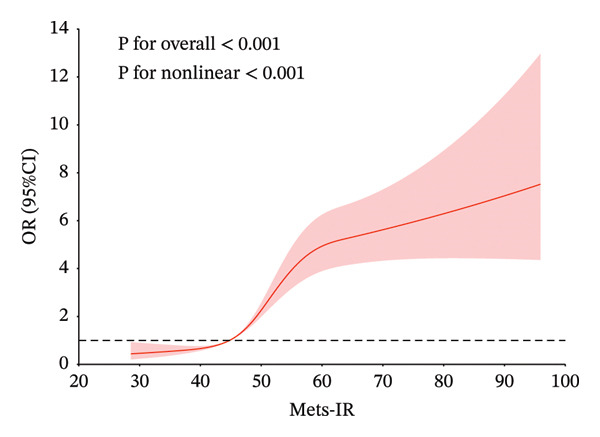
(c)

**Figure:**
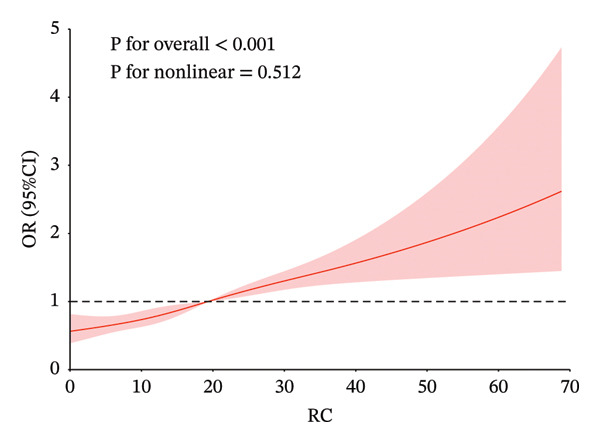
(d)

Table 5 shows the best cutoff points (234.49, 2.55, and 64.10) of TyG–BMI, TG/HDL‐c, and Mets‐IR, which are nonlinearly related to the metabolic syndrome, by the likelihood ratio test. When the above three indices are below the cutoff points, the risk of metabolic syndrome increases with the increase in the indices. There is no significant association between the continued increase in the indices above the cutoff points and the risk of metabolic syndrome.

**TABLE 5:** Threshold effect analysis of IR‐related indices and metabolic syndrome.

Variables	(95% CI)	*p*
TG/HDL‐c		
< 2.55	2.29 (1.98, 2.65)	< 0.0001
≥ 2.55	1.00 (0.89, 1.13)	0.9854
Likelihood ratio		< 0.001
TyG–BMI		
< 234.49	1.03 (1.03, 1.04)	< 0.0001
≥ 234.49	0.99 (0.99, 1.00)	0.1128
Likelihood ratio		< 0.001
Mets‐IR		
< 64.10	1.11 (1.10, 1.13)	< 0.0001
≥ 64.10	0.98 (0.96, 1.00)	0.0705
Likelihood ratio		< 0.001

*Note:* Adjustment variables: sex, age, education level, married status, frequency of meals, smoking, drinking, creatinine, and C‐reactive protein.

### 3.5. The Relationship Between Dynamic Changes in the IR‐Related Index and Metabolic Syndrome

Table 6 reveals the relationship between IR‐related index changes and the incidence of metabolic syndrome. Using the low–low group as a reference, in terms of Mets‐IR, both low–high and high–high increase the risk of metabolic syndrome (OR = 4.89, 95%CI: 3.26, 7.34; OR = 9.87, 95%CI: 7.08, 13.74). There is no statistical difference in the risk of metabolic syndrome when Mets‐IR changes from high to low (OR = 0.64, 95%CI: 0.28, 1.44, *p* = 0.281). TyG–BMI and RC have a similar impact on the metabolic syndrome as Mets‐IR. When TG/HDL‐c changes from low to high or high to high (OR = 8.16, 95%CI: 5.72, 11.66; OR = 12.58, 95%CI: 8.96, 17.66), its impact on the incidence of metabolic syndrome is similar to TyG–BMI and Mets‐IR. However, high–low in TG/HDL‐c could also increase the incidence of metabolic syndrome (OR = 1.73, 95%CI: 1.02, 2.92, *p* = 0.0412).

**TABLE 6:** Relationship between dynamic changes in the IR‐related indices and metabolic syndrome.

Variables	Model 1	*p*	Model 2	*p*	Model 3	*p*
Mets‐IR						
Low–low	1.00 (Reference)		1.00		1.00	
Low–high	4.55 (3.05, 6.78)	< 0.001	4.83 (3.22, 7.25)	< 0.001	4.89 (3.26, 7.34)	< 0.001
High–low	0.76 (0.36, 1.58)	0.457	0.63 (0.28, 1.43)	0.274	0.64 (0.28, 1.44)	0.281
High–high	8.47 (6.14, 11.67)	< 0.001	9.68 (6.96, 13.47)	< 0.001	9.87 (7.08, 13.74)	< 0.001
RC						
Low–low	1.00 (Reference)		1.00		1.00	
Low–high	3.34 (2.57, 4.34)	< 0.001	3.13 (2.40, 4.07)	< 0.001	3.11 (2.39, 4.05)	< 0.001
High–low	0.60 (0.34, 1.07)	0.083	0.62 (0.35, 1.10)	0.104	0.62 (0.35, 1.09)	0.097
High–high	4.54 (3.47, 5.95)	< 0.001	4.42 (3.37, 5.81)	< 0.001	4.42 (3.37, 5.81)	< 0.001
TyG–BMI						
Low–low	1.00 (Reference)		1.00		1.00	
Low–high	5.31 (3.50, 8.05)	< 0.001	5.89 (3.86, 8.98)	< 0.001	5.84 (3.82, 8.93)	< 0.001
High–low	1.39 (0.76, 2.54)	0.289	1.27 (0.67, 2.39)	0.467	1.27 (0.67, 2.39)	0.469
High–high	10.11 (7.14, 14.30)	< 0.001	11.26 (7.89, 16.08)	< 0.001	11.25 (7.86, 16.09)	< 0.001
TG/HDL‐c						
Low–low	1.00 (Reference)		1.00		1.00	
Low–high	8.17 (5.74, 11.62)	< 0.001	8.28 (5.80, 11.82)	< 0.001	8.16 (5.72, 11.66)	< 0.001
High–low	1.76 (1.05, 2.94)	0.032	1.74 (1.03, 2.94)	0.039	1.73 (1.02, 2.92)	0.041
High–high	11.68 (8.35, 16.33)	< 0.001	12.78 (9.10, 17.93)	< 0.001	12.58 (8.96,17.66)	< 0.001

*Note:* Trends in the dynamics of the indices. Low–low: low status at baseline, low status at end of follow‐up. Low–high: low status at baseline, high status at the end of follow‐up. High–low: high status at baseline, low status at the end of follow‐up. High–high: high status at baseline, high status at the end of follow‐up. Model 1 is a crude model. Model 2 is adjusted for age, gender, education level, married status, and frequency of meals. Model 3 is additionally adjusted for smoking, drinking, creatinine, and C‐reactive protein.

## 4. Discussion

In this prospective cohort study, we research the relationship between four indices for assessing IR and the risk of metabolic syndrome. In terms of predictive ability, TyG–BMI (AUC = 0.703) and Mets‐IR (AUC = 0.702) are the better predictive indices for metabolic syndrome. Regarding changes in the indices, low–low as a reference, both low–high and high–high changes in all indices could increase the risk of metabolic syndrome. In terms of TG/HDL‐c, high–low could also increase the incidence of metabolic syndrome compared to the low–low group. For the other indices, the risk of metabolic syndrome does not increase as long as the index is reduced to the low state.

A previous cross‐sectional study shows recognition of metabolic syndrome by 13 types of obesity and metabolism‐related indices, which find that TyG‐related parameters have the strongest ability to identify metabolic syndrome in the cross section (AUC: 0.853–0.924) [25]. Compared with VAI, LAP, and TG/HDL‐c, another study also finds that TyG‐related parameters are the strongest indicators for assessing IR [26]. The above studies show differences in the prediction of IR and metabolic syndrome by different indices, and IR is a central pathophysiologic mechanism of the metabolic syndrome. Therefore, we conduct this prospective cohort study to compare the predictive ability of different IR‐related indices for metabolic syndrome and the effect of dynamic changes on the metabolic syndrome.

Our results show that TyG–BMI and Mets‐IR have the largest AUC value compared to the other indicators. Several reasons contributed to explaining these results: Firstly, TyG and TyG‐related parameters could better assess the degree of individual IR than HOMA‐IR [27, 28]. Secondly, in the calculation of the index, TyG–BMI and Mets‐IR combine fasting glucose and TGs from the diagnostic criteria for metabolic syndrome. The results are better than the findings which Lin et al. find that TyG has a good ability to predict the metabolic syndrome over a 5‐year period (AUC = 0.674) [29]. The study by M. Mirr et al. also demonstrates that the TyG‐related parameter has a better ability to predict the metabolic syndrome than TyG [30]. Thus, focusing on TyG–BMI and Mets‐IR in middle‐aged and older adults could contribute to better early prediction of metabolic syndrome. In addition, we find that RC, which is considered related to IR, is poorly predictive of metabolic syndrome.

The present study explores the risk range of IR‐related indices for metabolic syndrome using restricted cubic splines. For IR‐related indices, studies have shown that the TyG index and HOMA‐IR have a linear relationship with metabolic syndrome [31]. However, for TG/HDL‐c, TyG–BMI, and Mets‐IR, the relationship between them and metabolic syndrome is a significant positive nonlinear correlation. Through restricted cubic splines and threshold effect analysis, we identify the cutoff point at which the index and the risk of metabolic syndrome show a significant upward trend and tend to stabilize. The values between two cutoff points may be considered as the risk range for metabolic syndrome. The relationship between RC and metabolic syndrome is significantly linear.

For the dynamic changes of these indices, we conclude that the risk of metabolic syndrome is elevated in both the low–high and high–high metabolic syndrome groups when compared to the low–low group. Our previous preprint also demonstrated that when IR indices rise to high‐level groups, the risk of metabolic syndrome increases [32]. In terms of TG/HDL‐c, referenced from low–low, high‐to‐low also increases the risk of metabolic syndrome. In terms of Mets‐IR, RC, and TyG–BMI, there is no statistical difference from high to low for the risk of metabolic syndrome. Thus, monitoring the dynamics of the IR index could contribute to predicting the metabolic syndrome. For the people who are currently at a high Mets‐IR, RC, or TyG–BMI, the risk of metabolic syndrome will not increase as long as the index value is decreased to the low state in the future. Thus, it is crucial to encourage people to maintain a low index status through diet and exercise, which will decrease the occurrence of metabolic syndrome. In terms of Mets‐IR and TyG–BMI, we find that the risk of metabolic syndrome is more than eight times higher in low–high, compared to the low–low. Therefore, for the people who are currently at a low Mets‐IR or TyG–BMI, it is important to prevent them from becoming high index people in the future.

RC is a newly proposed index in recent years that has been proven to be associated with coronary heart disease and diabetes mellitus [33]. The elevation of RC could cause a high cholesterol environment, which reduces the survival of pancreatic beta‐cells, leading to reduced insulin secretion [34]. In addition, it has been suggested that RC may cause IR and inflammatory states potentially [35]. The present study shows that RC is a slightly poorer predictor of metabolic syndrome and more research is needed on the role of RC in the metabolic syndrome.

### 4.1. Strengths and Limitations

The present study finds that IR‐related indices are associated with the development of metabolic syndrome and compares the differences in predicting the occurrence of metabolic syndrome with these indices. We further find that the dynamic pattern of IR‐related indices is associated with the development of future metabolic syndrome. These recognized IR‐related indices are clinically accessible. This study uses data from CHARLS to analyze the relationship between metabolic syndrome and IR‐related indices in middle‐aged and elderly Chinese. CHARLS is a high‐quality microdatabase representing Chinese households and individuals aged 45 years and older. There are some limitations in the present study that need to be identified. Firstly, as this study is a secondary analysis based on follow‐up survival and participants who completed follow‐up assessments, it may be subject to present‐incidence (Neyman) bias. This occurs when individuals experiencing outcomes early or those lost to follow‐up are more likely to be excluded, potentially leading to underestimation or overestimation of the association between exposure and metabolic syndrome. Secondly, because of the need for blood test results, data from 2011 to 2015 in CHARLS are selected for this cohort, with a follow‐up period of 4 years. This study mainly focuses on comparing the predictive ability of four IR‐related indices for metabolic syndrome and the relationship between their dynamic changes and the risk of metabolic syndrome. More studies need to be conducted to clarify the relationship between dynamic changes in other indices and the metabolic syndrome. Thirdly, for the dynamic changes in indices, we only collect the change between two points in time and do not know the status of the change in the index before the baseline. The following studies could collect indexes at more time points to analyze the relationship between different types of changes in the indices and the metabolic syndrome.

## 5. Conclusions

TyG–BMI and Mets‐IR could be better indices for predicting the occurrence of metabolic syndrome in middle‐aged and elderly populations. For individuals with indices below the cutoff points, it is advisable to avoid an increase in IR‐related indices to prevent metabolic syndrome. A dynamic variety of these indices, including TG/HDL‐c, Mets‐IR, TyG–BMI, and RC, could predict the higher risk of the incidence of metabolic syndrome. Monitoring the dynamic changes in the above IR indices could contribute to preventing the occurrence of metabolic syndrome in middle‐aged and elderly populations.

**Nomenclature**
BMI: Body mass index
WC: Waist circumference
DBP: Diastolic blood pressure
SBP: Systolic blood pressure
CRP: C‐reactive protein
TG: Triglyceride
TC: Total cholesterol
IR: Insulin resistance
HDL‐c: High‐density lipoprotein cholesterol
Mets‐IR: Metabolic score for insulin resistance
TG/HDL‐c: Triglyceride/ high‐density lipoprotein cholesterol
TyG–BMI: Triglyceride–glucose index with body mass index
RC: Remnant cholesterol

## Author Contributions

Xinfeng Li (first author): data curation, formal analysis, methodology, writing–original draft. Xiaohui Li: data curation, and formal analysis. Chifa Ma: data curation, and methodology. Chenfei Li: formal analysis and methodology. Mingxia Yuan (corresponding author): project administration and writing–review and editing.

## Funding

No external funding was provided for this study.

## Ethics Statement

This study follows the principles outlined in the Declaration of Helsinki and receives approval from the Biomedical Ethics Review Committee of Peking University (IRB00001052‐11015). All participants sign informed consent forms.

## Consent

The authors have nothing to report.

## Conflicts of Interest

The authors declare no conflicts of interest.

## General Statement

Due to the previous submission, this manuscript has been submitted as a preprint [Xinfeng Li, Xiaohui Li, Chifa Ma et al. (2024). The Relationship Between Dynamic Changes in The Insulin Resistance Related Indexes and Metabolic Syndrome in Middle‐aged and Elderly Population. Preprint, researchsquare.com] https://doi.org/10.21203/rs.3.rs-4422926/v1. Some of the results in this manuscript were consistent with those in the preprint. Compared with the preprint, this manuscript further elaborated the relationship between insulin resistance indices and metabolic syndrome. Furthermore, we have provided a more detailed description of the methodology, figures and tables, and discussion sections within this manuscript. We confirm that this manuscript is the final version. All authors read and approved the final manuscript.

## Supporting Information

The document presents research data concerning metabolic syndrome and insulin resistance indices.

## Supporting information


**Supporting Information** Additional supporting information can be found online in the Supporting Information section.

## Data Availability

The data of this study are open to the academic community on the following website: http://CHARLS.pku.edu.cn/en, accessed on May 9, 2024.
